# The human metabolic reconstruction Recon 1 directs hypotheses of novel human metabolic functions

**DOI:** 10.1186/1752-0509-5-155

**Published:** 2011-10-01

**Authors:** Ottar Rolfsson, Bernhard Ø Palsson, Ines Thiele

**Affiliations:** 1 Center for Systems Biology, University of Iceland, Sturlugata 8, 101 Reykjavik, Iceland; 2 Faculty of Industrial Engineering, Mechanical Engineering & Computer Science, University of Iceland, 101 Reykjavik, Iceland

## Abstract

**Background:**

Metabolic network reconstructions formalize our knowledge of metabolism. Gaps in these networks pinpoint regions of metabolism where biological components and functions are "missing." At the same time, a major challenge in the post genomic era involves characterisation of missing biological components to complete genome annotation.

**Results:**

We used the human metabolic network reconstruction RECON 1 and established constraint-based modelling tools to uncover novel functions associated with human metabolism. Flux variability analysis identified 175 gaps in RECON 1 in the form of blocked reactions. These gaps were unevenly distributed within metabolic pathways but primarily found in the cytosol and often caused by compounds whose metabolic fate, rather than production, is unknown. Using a published algorithm, we computed gap-filling solutions comprised of non-organism specific metabolic reactions capable of bridging the identified gaps. These candidate solutions were found to be dependent upon the reaction environment of the blocked reaction. Importantly, we showed that automatically generated solutions could produce biologically realistic hypotheses of novel human metabolic reactions such as of the fate of iduronic acid following glycan degradation and of N-acetylglutamate in amino acid metabolism.

**Conclusions:**

The results demonstrate how metabolic models can be utilised to direct hypotheses of novel metabolic functions in human metabolism; a process that we find is heavily reliant upon manual curation and biochemical insight. The effectiveness of a systems approach for novel biochemical pathway discovery in mammals is demonstrated and steps required to tailor future gap filling algorithms to mammalian metabolic networks are proposed.

## Background

An *in silico *model of a genome-scale metabolic network reconstruction is based upon a biochemically, genetically and genomically (BiGG) structured knowledge base [1,2]. It is subject to research that, in many cases, entails predicting an organism's phenotypic response to gene deletions and/or environmental perturbations *in silico*. These properties have resulted in widespread applications of metabolic models in microbial bioengineering, contextualisation of high-throughput data, and biochemical pathway discovery [2,3]. While the number of microbial genome-scale metabolic networks has increased exponentially over the past 10 years [4], fewer have been reconstructed for higher eukaryotes as their inherent complexity results in larger and more complex models which are harder to experimentally validate [5,6]. To date, only mouse [7-9], bovine [10], and human [11-13] genome-scale metabolic networks have been reconstructed. These latter ones have successfully been applied to systems driven eukaryotic metabolic research. For example, the human genome-scale metabolic network RECON 1 has been used to reveal transcriptional regulatory signatures of type 2 diabetes [14], to create tissue-specific models [15], to predict drug-off target effects [16], and to simulate cell specific metabolic changes upon pathogen infection [17]. Their potential to discover novel metabolic functions has however not been demonstrated.

**Figure 1:**
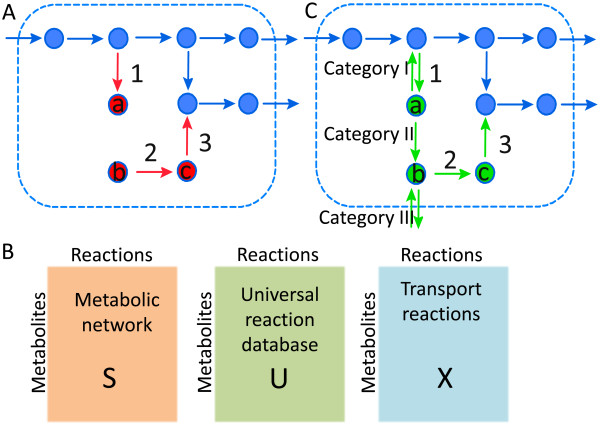
**Automated gap filling of RECON 1 using the SMILEY algorithm**. **A) **A simplified metabolic network. Reactions that are able to carry flux are shown in blue. Reactions unable to carry flux (red) are blocked and are caused by a root no-consumption metabolite (a in 1) and a root no-production metabolite (b in 2). Dead end metabolites can cause multiple blocked reactions refferred to as a cascade of blocked reactions. Reactions 2 and 3 occur in a blocked cascade caused by b. Note that c is a blocked intermediate but not a root-no production metabolite. **B) **The SMILEY algorithm identified reactions in the metabolic reaction matrix **S **(e.g. RECON 1) that were unable to carry flux under steady state conditions and then computed resolving reactions found in either **U **or **X **that needed to be added to **S **in order to restore flux through the blocked reaction. **C) **SMILEY solutions were categorised based on the resolving reactions required to restore flux. A category I reversal solution, if added to the network shown in A, only restores flux through reaction 1. The category II solution, addition of a novel metabolic reaction, restores flux through reactions 1, 2 and 3. The category III transport solution restores flux through reactions 2 and 3 only. SMILEY can suggest multiple solutions for a blocked reaction.

RECON 1 accounts for 1496 ORFs, 2004 proteins, 2766 metabolites, and 3311 metabolic reactions. It represents one of the most comprehensive biological network reconstructions published to date [11]. RECON 1 is bipartite in nature. First, it is a BiGG knowledge base containing information on the components of human metabolism, mined by extensive literature review [4] and accessible online at http://bigg.ucsd.edu[18]. Second, it is a mathematical model in which reactions and their metabolites are presented in matrix format using metabolite stoichiometries. RECON 1 is therefore also a stoichiometric matrix amenable to constraint-based analysis using linear programming as the reaction stoichiometries impose constraints on the flow of metabolites through the network. As an additional constraint, a quasi steady state is assumed, meaning that the total amount of any metabolite being produced must equal the total amount of that metabolite being consumed. Using constraint-based analysis, the flow of metabolites through the network reactions can be calculated. In addition, and perhaps most importantly, reactions can be identified which cannot carry flux due to metabolites being either only produced or consumed in the network [4,19]. These latter reactions and metabolites represent gaps in the metabolic network and are referred to as blocked reactions and dead-end metabolites, respectively. Dead-end metabolites can result in multiple blocked reactions, referred to as blocked reaction cascades. Consequently they have been further characterised as root-no production or root-no consumption metabolites to distinguish them from other blocked metabolites within the blocked cascade [20] (Figure 1a). In this manner RECON 1 can be utilised to pinpoint parts of metabolic pathways where knowledge is incomplete. A recent investigation highlighted that novel biochemical reactions can still be uncovered in the central metabolism of *E. coli *by employing a systems approach [21]. Considering that 30-50% of all known enzyme activities are not associated with genes [22-24] and over 50% of all genes in higher organisms are not associated with protein function [25], it appears that many human biological processes have yet to be discovered. Enzymes with no known genes (e.g. orphan enzymes) and genes of unknown function underlie metabolic network gaps that manifest themselves as blocked reactions and dead-end metabolites in RECON 1. As the function and/or substrate specificity of many known human enzymes has not been fully elucidated, these similarly serve as candidates for filling network gaps.

Multiple computational algorithms have been devised to address gaps in metabolic network models in an automated manner [26]. These methods utilise metabolic network analysis, such as flux variability analysis (FVA) [27], in order to identify network gaps, alongside comparative genomics/metabolomics to suggest candidate metabolic reactions capable of restoring flux through the blocked reaction and/or dead-end metabolite. Experimental data, such as organism growth profiles [28,29] or metabolic flux data [30] can be integrated with metabolic models in order to highlight model gaps, although this is not always a requirement [26]. One such algorithm is the SMILEY algorithm, which has been successfully used to uncover novel metabolic functions required to explain discrepancies between the observed and model predicted growth phenotypes of *E. coli *on various substrates [28].

Because of the difference between humans and microorganisms in terms of the number of related organisms for which biological, biochemical, and genetic information exists, it was not known whether an automated approach to gap-filling of RECON 1 could yield biologically plausible hypotheses. Here we investigated the potential of RECON 1 for discovery of novel reactions involved in human metabolism. We used FVA to identify dead-end metabolites and blocked reactions in RECON 1 and SMILEY to propose reactions capable of restoring flux through the identified dead-end metabolites. We then characterised the metabolic pathway distribution of identified gaps and their solution types in order to get an idea of how gaps are distributed within RECON 1 and in what manner these gaps are resolved by SMILEY. Finally we validated the automatically generated metabolic reaction hypotheses by manual literature review in order to assess the biological relevance of the proposed solutions with the goal of identifying suitable experimental targets.

## Results

In this study we first identified blocked reactions and dead-end metabolites occurring in RECON 1 using FVA [27]. We then employed SMILEY [28] to compute reactions that could be added to the RECON 1 (**S**) from universal reaction databases (**U**, **X**) to enable flux through a blocked reaction (Figure 1b). The matrix **U **was compiled from an extensive list of known metabolic reactions obtained from the KEGG database [31] while the matrix **X **contained transport reactions in and out of the system for every metabolite contained within **S **and **U**. SMILEY therefore proposed what reactions, irrespective of organism, needed to be added to RECON 1 in order to fill a network gap. If none were identified, SMILEY suggested transport of the dead-end metabolite into or out of the cell. Up to twenty solutions were suggested for each knowledge gap and each solution could be composed of multiple resolving reactions. Note that the number of solutions returned by SMILEY is user defined. Inspired by Satish Kumar et al. [20] we split the computed solutions into three categories based upon whether the complete solution involved a reversal of the directionality of the blocked reaction, addition of novel reaction(s), or addition of a transport reaction, defined as category I-III type solutions respectively (Figure 1c). The next four sections deal with the characterisation and metabolic pathway distribution of the gaps that we addressed in RECON 1 while the remainder of the results chapter reports analysis of SMILEY solutions and specific case studies.

### Gap analysis basis

**Figure 2:**
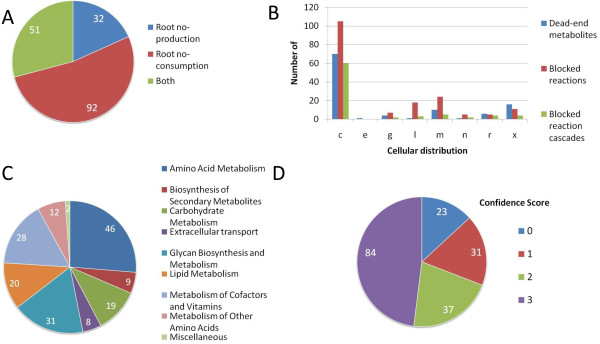
**Characterisation of blocked reactions in RECON 1**. **A) **Classification of blocked reactions in RECON 1 depending on their causative dead-end metabolite. **B) **The cellular distribution of dead-end metabolites, blocked reactions, and blocked reaction cascades within cellular compartments accounted for in RECON 1. **C) **The metabolic pathway distribution of the 175 blocked reactions investigated. **D) **The distribution of BiGG database confidence scores for the blocked reactions investigated. 3 = biochemical and or genetic evidence, 2 = physiological evidence or evidence from a nonhuman mammalian cell, 1 = modelling evidence, 0 = unevaluated.

We identified 175 blocked reactions in 80 reaction cascades that were caused by 109 dead-end metabolites found in RECON 1. These numbers corresponds to 5% and 4% of the total number of reactions and metabolites accounted for in RECON 1, respectively. We observed that over half of the blocked reactions were caused by root no-consumption metabolites while roughly a quarter were due to root no-production metabolites with the remainder caused by both types of dead-end metabolites (Figure 2a). Reactions that have both types of dead-end metabolites represent metabolic reactions which are entirely uncoupled from the metabolic network, and whose effect on global metabolism can therefore not be described accurately by the metabolic model.

### Sub-cellular distribution of gaps

The sub-cellular distribution of the dead-end metabolites and blocked reactions showed that the majority were found in the cytosol with the remainder distributed within the various cellular compartments, most notably in the lysosome, mitochondria, and peroxisome (Figure 2b). This observation agrees with the distribution of dead-end metabolites found in the eukaryotic metabolic reconstruction iND750 for *Saccharomyces cerevisiae *[20,32]. Because some of the dead-end metabolites were responsible for multiple blocked reactions, which themselves could be associated with both types of dead-end metabolite, there was not a direct relationship between the number of dead-end metabolites and the number of blocked reactions within cellular compartments. In addition, blocked reactions can take part in metabolic subsystems/reaction cascades that span more than one cellular compartment. For example, in the mitochondria we found 24 blocked reactions participating in six distinct reaction cascades and ten dead-end metabolites. However, only seven of the dead-ends were responsible for the 24 blocked reactions while the remaining three metabolites caused blocked reactions in the cytosol. Gap distributions can be better understood by looking at the distribution of blocked reaction cascades caused by the dead-end metabolites. These data suggest that the number of knowledge gaps in cellular compartments other than the cytosol is around two to five knowledge gaps per compartment (Figure 2).

### Metabolic pathway distribution of gaps

The metabolic pathway distribution of the blocked reactions is shown in Figure 2C and reflects their sub-cellular distribution. All of the metabolic pathways shown had blocked reactions in the cytosol. Most of the blocked reactions were observed in amino acid metabolism with the remainder distributed within the metabolic pathways shown. Of those involved in amino acid metabolism, nearly half were part of multiple reaction cascades in tryptophan metabolism (Additional file 1). These reactions were blocked due to root no-consumption heterocyclic derivatives of tryptophan, such as anthranilate, kynurenic acid, 5-methoxy-indole acetate, and more, suggesting missing information on the metabolic fate of these compounds. Apart from knowledge gaps in tryptophan metabolism, blocked reactions were also identified in metabolic subsystems associated with eleven of the twenty most common amino acids. These gaps were due to single blocked reactions or reaction cascades implying that current information concerning the metabolism of these amino acids is fairly complete.

Multiple blocked reactions were also observed in glycan biosynthesis, the metabolism of cofactors/vitamins and lipid metabolism. As opposed to those observed in amino acid metabolism, these blocked reactions were involved in relatively few reaction cascades. Many of the blocked reactions in glycan metabolism, for example, were part of two reaction cascades involved in the degradation of heparan sulfate and dermatan sulfate in the lysosome, respectively. The root no-consumption metabolite glucose-1, 3-mannose and derivatives thereof were also the cause of several blocked reactions in glycan biosynthesis. Many blocked reactions involved in the metabolism of cofactors/vitamins took place in the mitochondria due to just two root no-consumption metabolites causing gaps in the biosynthesis of ubiquinone and vitamin D. Similarly, multiple reactions in lipid metabolism were blocked in glycerophospholipid biosynthesis in the cytosol due to the root no-consumption metabolite plasmalogen in the cytosol. In total, 32% of the knowledge gaps investigated were associated with two or more reactions. Finding a solution to the dead-end metabolites in these reaction cascades could therefore result in the connection of multiple blocked reactions back into the metabolic network. The majority of blocked reactions were not, however, part of reaction cascades (Additional file 2).

### Knowledge status of reactions causing gaps

We investigated whether the gaps addressed in this study were correlated with a lack of information available for each blocked reaction by assessing their confidence scores. Each reaction in RECON 1 has an assigned confidence score that allows the experimental evidence underlying the reaction to be quickly assessed [11]. Figure 2D shows the distribution of confidence scores of the reactions investigated. Roughly two thirds of the blocked reactions were supported by biochemical or physiological evidence, which is similar to what is observed for all reactions contained in RECON 1 [11]. This distribution implies that the addressed knowledge gaps are not simply due to ill-defined metabolic reactions. Rather, the fate of the participating metabolites within the metabolic network or how they contribute to human metabolism is not known. In order to suggest plausible hypotheses of how this might take place, we investigated whether the blocked reactions could be circumvented or connected back into the RECON 1 using reactions found in the KEGG database [31] in an automated manner by running the SMILEY algorithm [28].

### Solutions to a blocked reaction are dependent upon the robustness of its metabolic network

**Figure 3:**
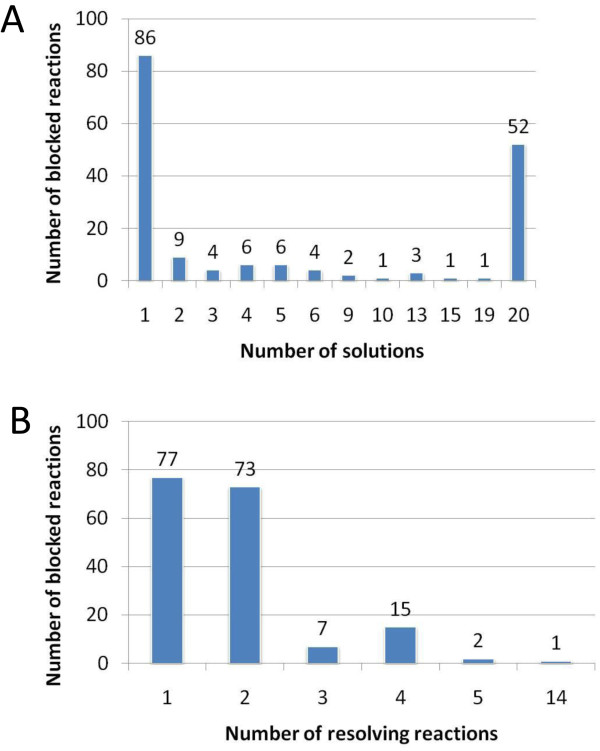
**Characterisation of gap filling solutions proposed by SMILEY**. **A) **The number of SMILEY solutions proposed for each blocked reaction was not even, suggesting that it is easier to incorporate some dead-end metabolites into RECON 1 over others. Approximately 49% of the blocked reactions had a single proposed solution while 29% could be circumvented in a highly dynamic manner with twenty proposed solutions. **B) **85% of the blocked reactions had SMILEY solutions composed of less than three resolving reactions.

The SMILEY algorithm suggested up to twenty solutions for each blocked reaction. Each solution fell into one of the three category types outlined in Figure 1. We first investigated how trivial it was to bypass the blocked reactions using reactions from the KEGG database (**U **matrix). Nearly half of the blocked reactions had more than one solution indicating that flux could be restored through these reactions in multiple ways (Figure 3a). Underlying this is that the more robust the non-organism specific reaction environment of the blocked reaction is, the more likely that the blocked reaction will have multiple solutions as there will be more known reactions capable of acting upon the blocked reaction components. For example, a blocked reaction caused by a common, well-characterized metabolite that is a component of multiple metabolic reactions will have multiple SMILEY solutions while the opposite is true for blocked reactions caused by less well characterized metabolites. The number of solutions obtained for a blocked reaction was independent of the metabolic pathway (Additional file 3). Blocked reactions in RECON 1 are therefore not caused only by dead-end metabolites whose global metabolic role is unknown, rather metabolites and reactions can be well characterized metabolic components of other organisms while there fate is not known in humans. The result complements our earlier observation that knowledge gaps are found in all metabolic pathways, independent of the confidence scores of the reactions making up the particular pathway.

### SMILEY solutions involve few resolving reactions

Inspection of the SMILEY output made it clear that assessing each of the 1335 SMILEY solutions represented a time consuming task. Our focus was to assess whether SMILEY could generate biologically relevant hypotheses of missing reactions in human metabolism rather than produce a detailed list of missing enzyme functionalities. We therefore decided to focus on the SMILEY solutions containing the least number of resolving reactions for each of the 175 blocked reactions hereafter referred to as S1 solutions.

**Figure 4:**
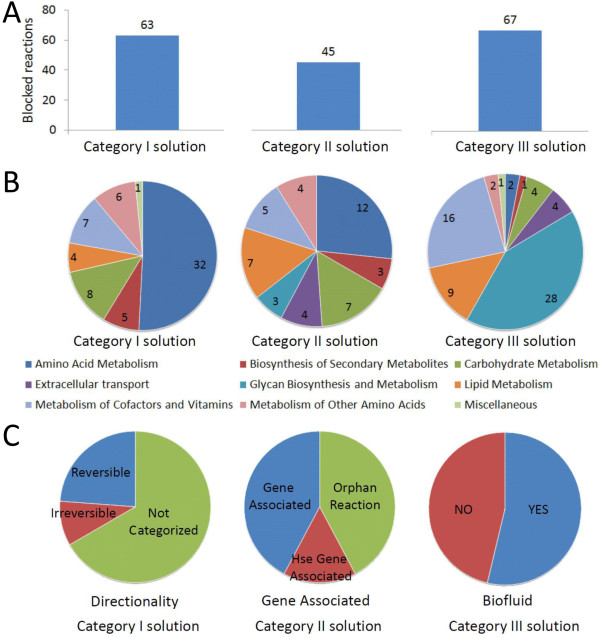
**Blocked reactions have different SMILEY solutions depending upon their metabolic origin and were validated with experimental evidence**. Blocked reactions were resolved by connecting their corresponding blocked metabolite back into RECON 1. **A **shows the number of blocked reactions resolved with each solution category and, directly below in **B**, the metabolic pathway distribution of the blocked reactions having that particular SMILEY solution. Some blocked reactions, such as those involved in amino acid metabolism, are easily bypassed using functionalities already described in the KEGG database. Others, such as those involved in glycan biosynthesis, can only be solved by transporting their causative dead-end metabolite out of the system. **C) **Proposed solutions were validated by comparing category I solutions to experimentally reported enzyme directionalities. Similarly, we investigated whether category II solutions were gene associated in humans and whether the dead-end metabolites, to which the category III solutions applied, have been detected in human biofluids.

Analysis of S1 solutions showed that flux could be restored through all the blocked metabolites by incorporation of relatively few resolving reactions. Interestingly, 85% of the blocked reactions were circumvented with just one or two resolving reactions (Figure 3b). We observed that this was due to the category of the SMILEY solutions (Figure 4). 74% of the blocked reactions had a category I or III solution, which in the majority of cases involved at most two resolving reactions. The remaining blocked reactions had category II solutions where novel reaction(s) had to be added in order to incorporate the blocked metabolite.

Mindful that we might overlook plausible solutions for blocked reactions having multiple SMILEY solutions by focusing entirely on S1 solutions, we investigated the categories of the alternative solutions. We found that, when available, alternative solutions were either of the same solution category as the S1 solution or were category III transport solutions (Additional file 4). This result indicated that the S1 solutions directly reflect the solution categories available for each blocked reaction although the resolving reactions comprising the solutions were different.

### Blocked reactions have different S1 solutions depending on metabolic origin

Roughly one third of all the blocked reactions were resolved with category I solutions (Figure 4). Nearly half of these applied to blocked reactions involved in amino acid metabolism (Figure 4B), many of which were part of tryptophan and lysine metabolism in the cytosol. With respect to their metabolic origin, up to 70% of the total number of blocked reactions originating from a particular metabolic pathway had category I solutions (Additional file 5). Notably, none of the blocked reactions involved in glycan biosynthesis and metabolism had category I solutions. Instead, these reactions were primarily resolved by category III transport solutions. These applied to 90% of the blocked reactions involved in glycan biosynthesis and metabolism, more specifically to those in chondroitin sulphate and heparan sulphate degradation in the lysosome and N-glycan biosynthesis in the golgi apparatus. Apart from glycan metabolism, category III solutions applied to multiple blocked reactions involved in the metabolism of cofactors and vitamins and in lipid metabolism. Few blocked reactions within amino acid metabolism and the biosynthesis of secondary metabolites had category III solutions. Category II solutions were the least common solution. With respect to their metabolic origin, 10-50% of the blocked reactions had this solution type (Additional file 5). Many of these applied to blocked reactions taking part in various metabolic subsystems of amino acid metabolism such as tyrosine metabolism and urea cycle metabolism. Multiple blocked reactions in carbohydrate metabolism and fatty acid metabolism also had category II solutions.

### Validation of SMILEY solutions

**Figure 5:**
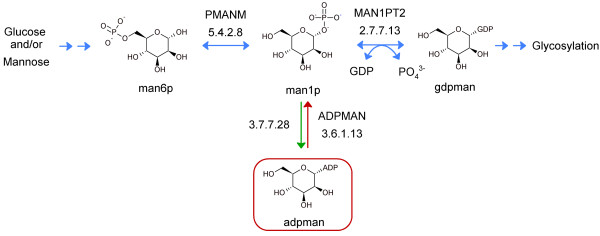
**Category I solution for the blocked reaction ADPMAN**. ADP-mannose (boxed in red) is a root no-production metabolite within RECON 1. The SMILEY solution (green arrow) involved reversal of the blocked reaction ADPMAN (red arrow). This appears plausible as an enzyme catalysing such a reaction (EC 3.7.7.28) has been described in various mammals [39] along with the finding that mannose-1-phosphate guanylyltransferase (EC 2.7.7.13) is known to be reversible and accept sugar donors other than GTP [40].

Category I and III solutions were only generated when no other solution was possible and therefore the blocked reactions that had these solutions contain metabolites whose global metabolic role has not been defined. This is however not a direct indicator of whether or not these solutions are biologically plausible. Category I reversal solutions to blocked reactions were validated by i) quantitative assignment of blocked reaction directionalities based on estimates of their Gibbs free energy changes [33] and ii) a query of experimentally reported reaction directionalities from the Brenda database [34]. Approximately 50% of the blocked reactions were calculated to be reversible while 23% were reported reversible in the Brenda database (Figure 4C and Additional file 6). Only four reactions were reported reversible by both methods which is due to the high number of the blocked reactions having unspecified reaction directionalities in the Brenda database (67%) and the uncertainties associated with reaction Gibbs free energy estimation due to unknown *in vivo *concentrations of the partaking metabolites [35]. These validation methods do not exclude reactions as reversible or not. Ultimately detailed inspection of each gap solution is required. Figure 5 shows the cytosolic reaction ADPMAN, which is catalysed by the nudix hydrolase NUDT5 (EC 3.6.1.13) and blocked in RECON 1 due to the root no-production metabolite ADP-mannose (adpman) [36-38]. The product, mannose-1-phosphate (man1p), is converted to GDP-mannose (gdpman) from either glucose or mannose and is ultimately utilised in N-glycan biosynthesis. A category I solution to ADPMAN was proposed by SMILEY. We found that, although the reversal of NUDT5 activity has not been reported, the conversion of man1p to adpman has been described in rats and cows (EC 2.7.7.28) [39]. Furthermore, the reaction MAN1PT2, catalysed by mannose-1-phosphate guanylyltransferase (EC 2.7.7.13), is known to accept both ATP and ITP in addition to GTP although with reduced activity [40]. This implies that formation of adpman from man1p, which is in effect a reversal of EC 3.6.1.13 activity, is plausible. The results suggest that reversing the directionality of blocked reactions to restore network flux can represent valid biological hypotheses although the biological fate of the dead end metabolite remains unknown.

**Figure 6:**
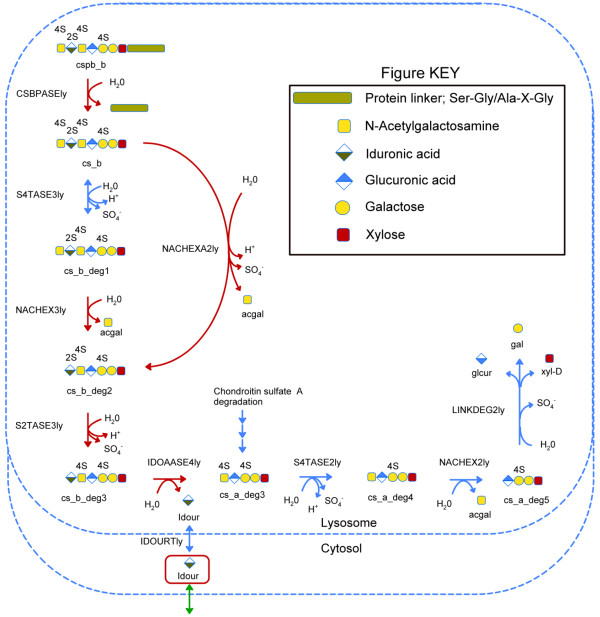
**Category III solution for five blocked reactions occurring in dermatan sulfate degradation**. Iduronic acid (idour, boxed in red) was identified as a root no-consumption metabolite in dermatan sulfate degradation in the lysosome. Flux was restored through the blocked reactions (red arrows) by addition of an extracellular transport function for idour (green arrow). Review of the literature indicates that this SMILEY generated hypothesis is biologically plausible. See text for details.

In order to validate the category III solutions, we investigated whether the metabolites to which the transport solutions applied have been detected in biofluids by querying the Human Metabolome Database [41]. The 67 blocked reactions that had category III S1 transport solutions were part of 24 reaction cascades. We found that 36 of the blocked reactions, part of eight reaction cascades, had blocked metabolites that have been detected in biofluids (Figure 4C). For example, we identified a cascade of five blocked reactions involved in dermatan sulfate degradation in the lysosome. Dermatan sulfate degradation is modelled in RECON 1 by the degradation of a glycoseaminoglycan chain of a defined length (Figure 6). Following initial cleavage of the polysaccharide from a serine residue at the reducing end of the core tetrasaccharide linkage by dermatan proteoglycan preotease, degradation occurs through a series of enzymatic steps involving desulfonation by sulfatases and subsequent hydrolysis of the glycosidic bonds connecting the monosacharide building blocks, iduronic acid and N-galactosamine, by exoglycosidases [42,43]. We found that dermatan sulfate degradation was blocked due to iduronic acid buildup in the cytosol following its cleavage from the dermatan polysaccharide in the reaction IDOAASE4ly (EC 3.2.1.76) and transport out of the lysosome in reaction IDOURtly [44-46]. The SMILEY solution for the blocked reaction IDOAASE4ly proposed transport of iduronic acid out of the cytosol in order to restore flux through the dermatan sulfate subsystem. These results suggest a transport function for iduronic acid out of the cytosol, which appears plausible as iduronic acid has been detected in urine (M. Fuller, personal communication; [43,47]. Iduronic acid was also identified as a root no-consumption metabolite in heparan sulfate degradation causing twelve blocked reactions. The transport solution for iduronic acid therefore resolves 18 blocked reactions occurring in glycan degradation. We found no reports of cell surface iduronic acid transporters in the literature. Detailed substrate specificity studies for human hexose transporters, however, have not been performed [48].

**Figure 7:**
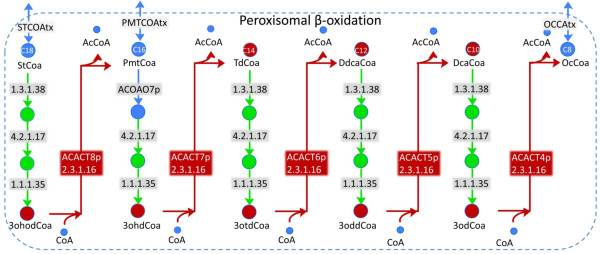
**Category II solution for blocked reactions in peroxisomal fatty acid degradation**. The blocked reactions (red arrows) are all catalysed by acetyl-CoA-acyltransferase (EC 2.3.1.16). The SMILEY solutions (green arrows) for each of the five blocked reactions generated a complete degradation pathway for saturated fatty acids in the peroxisome starting with stearoyl-CoA (StCoa) and ending in octanoyl-CoA (OcCoa) through stepwise removal of two carbons from the acyl chain. Abbreviations are as described in the BiGG database [18]. Enzyme commission numbers of all reactions are shown. Metabolites and reactions in blue are not blocked. The number of carbons of each fatty acid-CoA derivative is indicated. The results show that the SMILEY algorithm is capable of generating plausible solutions to blocked reactions in human metabolism. The resolving reactions suggested by SMILEY correspond to well-characterised reactions involved in the β-oxidation of fatty acids [50,79].

As opposed to category I and III SMILEY solutions, category II solutions involve adding resolving reactions to RECON 1 to restore flux through the dead-end metabolites. Of the 45 blocked reactions with category II solutions we found that seven had solutions composed of resolving reactions associated with human genes. Of those, five were part of peroxisomal beta-oxidation of long chain unsaturated fatty acids (Figure 7). These blocked reactions were all catalysed by 3-ketoacyl coenzyme A thiolase (EC 2.3.1.16), an enzyme responsible for the last step of the four recurring steps of fatty acid β-oxidation [49]. The SMILEY solutions successfully coupled the root no-production metabolites of these reactions, involving 3-oxo fatty acid coenzyme A derivatives, to their corresponding fatty acid in three resolving reactions. For example, the blocked reaction ACACT4p that forms octanoyl coenzyme A (ocCoA) from the root no-production metabolite 3-oxodecanoyl-CoA (3odCoA) was coupled to decanoyl-CoA (dcaCoA) through its stepwise oxidation by trans-2-enoyl-CoA reductase (EC 1.3.1.38), enoyl-CoA hydratase (EC 4.2.1.17), and 3-hydroxyacyl-CoA dehydrogenase (EC 1.1.1.35). Peroxisomal β-oxidation of unsaturated long chain fatty acids has not been completely elucidated in humans. However, it has been shown to generate ocCoA which is then transported to the mitochondria where it can be further oxidised and coupled to ATP production [50] as suggested by SMILEY. Although these results do not add to current knowledge of metabolism as these gaps result from incomplete modelling in RECON 1 of β-oxidation in the peroxisome, they suggest that SMILEY is capable of generating biologically realistic results to gaps in RECON 1.

**Figure 8:**
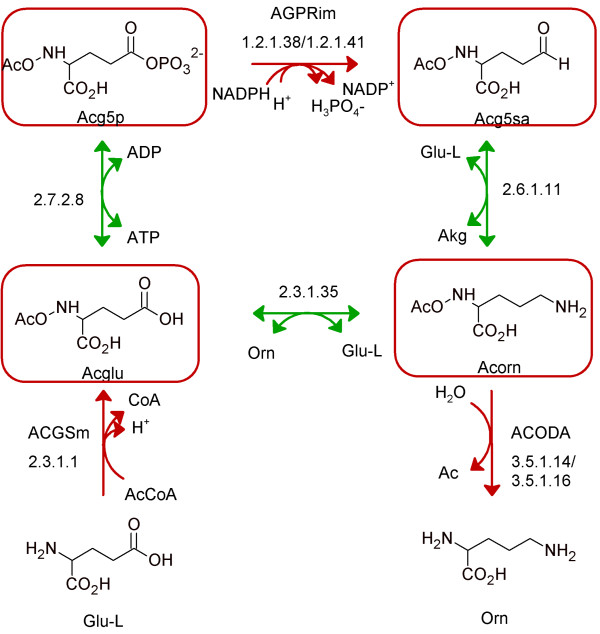
**Category II solutions to three blocked reactions in urea metabolism**. Flux was restored through the blocked reaction AGPRim by the addition of two resolving reactions (EC 2.7.2.8 and EC 2.6.1.11) which couple AGPRim to the blocked reactions ACGSm and ACODA through the consumption and production of their dead-end metabolites, N-acetylglutamate and N-acetylornithine, respectively. We found no evidence that could rule out this gap filling hypotheses. The S1 solutions to the blocked reactions ACGSm and ACODA were identical and involved inter-conversion of the dead-end metabolites of these reactions by glutamate N-acetyltransferase (EC 2.3.1.35). As opposed to the solution for AGPRim, we did not find any indications that reactions similar to EC 2.3.1.35 are found in humans.

The majority of category II solutions were composed of resolving reactions not associated with any known genes (19 solutions) or resolving reactions associated with enzyme activities encoded for by non-human genes (19 solutions) (Figure 4C). These represented the most exciting SMILEY solutions as they have the potential to unearth novel human metabolic enzymes. Figure 8 shows an example of category II solutions to three blocked reactions in urea metabolism. The SMILEY solution to AGPRim (EC 1.2.1.41), involves formation of its root no-production metabolite N-acetylglutamate-5-phosphate (acg5p) from N-acetylglutamate (acglu) and production of N-acetylornithine (acorn) from the root no-consumption metabolite N-acetylglutamate-5-semialdehyde (acg5sa). Acglu and acorn are themselves root no-consumption and root no-production metabolites in the blocked reactions ACGSm (EC 2.3.1.1) and ACODA (EC 3.5.1.16) respectively. Interestingly, the two resolving reactions proposed for AGPRim, catalysed by acetylglutamate kinase (EC 2.7.2.8) and acetylornithine transaminase (EC 2.6.1.11), therefore restore flux through all three blocked reactions. These reactions are part of the arginine biosynthesis pathway in prokaryotes and plants. They have not been associated with human genes, as arginine biosynthesis in mammals has been shown to occur through glutamate rather than N-acetylglutamate [51,52]. Human enzymes with similar functionalities have however been described. For example, human pyrroline-5-carboxylate synthase (P5CS) encodes glutamate-5-semialdehyde dehydrogenase activity (EC 1.2.1.41) and glutamate kinase activity (EC 2.7.2.11). P5CS is therefore a target candidate to catalyse a reaction where acg5sa is produced from acglu. Similarly, while acetylornithine transaminase activity (EC 2.6.1.11) has not been reported in humans, ornithine transaminase activity (EC 2.6.1.13) has [53]. A literature search did not reveal whether human ornithine transaminase is capable of accepting acorn as a substrate. However, ornithine transaminase from *Plasmodium vivax *shows 42-53% sequence similarity to eukaryotic ornithine transaminases and is known to accept both acorn and orn [54]. We suggest that following manual validation the hypotheses generated by SMILEY can guide experimental laboratory research. For selected blocked reactions, this is currently underway in our laboratory.

## Discussion

The investigation of large complex systems on a global scale makes it impractical and maybe even impossible to know details about all involved metabolites, genes, and proteins. At the same time, a high level of knowledge about metabolic subsystems and or enzyme activities is necessary in order to come up with hypotheses of particular metabolic fates and novel reactions. In this study we used a systems biology approach to characterise and fill gaps in human metabolism. The key results include i) many dead-end metabolites affect reaction cascades, ii) computationally predicted solutions require thorough manual curation and biochemical insight, and iii) four biological plausible hypotheses were identified. This work highlights that finding gene candidates for metabolic functions in the human genome is not a trivial issue and the extensive manual effort to curate the computational predictions of candidate reactions highlight the overall quality and quantity of data included in Recon 1.

We characterised knowledge gaps of human metabolism, represented by blocked reactions and dead-end metabolites identified in RECON 1 [11], to which solutions, in the form of non-organism specific metabolic reactions, could be found using the computer algorithm SMILEY [28] (Figure 1). We identified 175 blocked reactions, 70% of which had high confidence scores, and observed that while they were unevenly distributed within human metabolic pathways, most were found in the cytosol (Figure 2). Furthermore, we found that they arose due to different dead-end metabolite types that were in some cases responsible for up to 14 blocked reactions. These properties are likely to affect how trivial it will be to address these knowledge gaps experimentally and suggest that the impact of resolving these gaps, both in terms of novel metabolic discovery and their influence on RECON 1, will be different. For example, determining the fate of a metabolite, which results in multiple blocked reactions, will have a different impact on the biological accuracy of RECON 1 than resolving a single blocked reaction. Nevertheless, a single blocked reaction could be of great interest as a candidate target for novel metabolic discovery as its components could represent a drug target and resolving the gap could have unforeseen effects on network robustness, i.e., human metabolism. Also, assaying cytosolic reactions will be more straightforward than determining the function of compartmentalised reactions. Subsequently, which knowledge gaps are chosen for experimental research is ultimately a human decision depending on research goals, biological novelty factors, ease of experimental validation, and underlying evidence of the knowledge gap's validity.

We highlighted four examples of missing knowledge in human metabolism (Figures 5, 6, 7 and 8) that resulted in biologically plausible hypotheses using a combined algorithmic and manual approach. The hypotheses were strengthened with published experimental data. In the case of iduronic acid (Figure 6), a major constituent of glycosamine glycans, we argued for a hypothesis that an extracellular transport reaction needs to be added to RECON 1. Although no direct evidence for such transport could be identified in the human genome, the existence of iduronic acid in human urine (M. Fuller, personal communication) suggests that a transporter may be a biologically plausible solution. Further evidence is that the build-up of iduronic acid in the lysosome has been linked to lysosomal storage disorder caused by defects in the sialic acid lysosomal transporter [55]. Similarly, defects in α-L-iduronidase (3.2.1.76), the exo-glycohydrolase that cleaves iduronic acid off the non-reducing end of dermatan sulfate and heparan sulfate are known to cause a different type of lysosomal storage disorder, called mucopolysacharideosis I [43]. Despite its apparent involvement in disease, the metabolic fate of iduronic acid is unknown. The present work highlights knowledge gaps in human metabolic processes, such as the fate iduronic acid, which in the context of investigating lysosomal storage disorders due to protein deficiencies, have not been relevant but are now required to generate a complete picture of human metabolism. Our gap filling examples showed that algorithms, such as SMILEY, can be used to direct hypotheses of novel functions in human metabolism. Nevertheless, a semi-automated approach was required to assist with the identification of plausible gap filling candidates for experimental verification.

Multiple gap finding and gap filling algorithms exist, including GapFind/GapFill [20] and GrowMatch [29], and the use of alternative algorithms will undoubtedly increase the number of possible hypotheses as they employ different heuristics and data sources (e.g., universal databases). This work does not provide a comprehensive list of possible gap-filling reaction solutions but rather assesses the use of (semi)-automated computational approaches for identifying and completing missing functions in human metabolism on a large-scale. We found that computational tools, such as SMILEY, do not necessarily suggest biologically plausible gap filling hypotheses. The generated hypotheses need to be evaluated in a manual, time-consuming manner, similar to the gap filing process employed during the reconstruction approach [4,26]. The search for novel functions is therefore only semi-automated. Automated algorithms could however be trained, based on manual effort, to prioritize or exclude certain types of solutions. In addition, approaches could be developed that incorporate methods to build hypotheses of genes associated with orphan reactions [56-60], which SMILEY does not directly do.

Identification of genes associated with biological plausible hypotheses as suggested by SMILEY was a major challenge. Relatively few knowledge gaps were resolved using known metabolic functions (Figure 3), and the solutions required detailed literature review such that homology, of what were often prokaryotic genes/proteins to possible human counterparts, could be assessed. In light of our results, we believe that existing automatic gap filling approaches for uncovering gene function will be of limited use for mammals. This limitation arises from a lack of phylogenetic information, which is extensively explored for annotating microbial genomes [58,61]. Although various homology databases exist for mammalian genomes covering up to seventy mammalian species [62], the majority of phenotypic, genetic, and biochemical studies have been performed using mice, and to a lesser extent, human cells. Information derived from these databases therefore originates from few organisms making them less useful for annotation purposes. Furthermore, co-expression analysis is used in microbes to determine genes with related function [63-65] and could serve as a strategy for gene finding in the human genome. However, analysis of regions of correlated transcription (RCT) in human and mouse identified both related and unrelated genes being co-expressed [66]. The majorities of RCT were not found in both human and mouse, which the authors explained with i) missing definition of homology and/or synteny, ii) no conserved pattern, and/or iii) physiological differences between human and mice. This example highlights the challenges associated with finding novel gene functions in the human genome using established methods from the bacterial world. Novel approaches may include the use of protein-protein interaction data [67-69], tissue-specific information [70,71] and disease information [72] combined with gap filling algorithms. In particular, the latter work [72] observed a high degree of correlation between known co-occurring (co-morbid) diseases in patients and flux-coupling of the reactions that are perturbed in association with each of the disease states. Flux coupled reaction sets [73], or perfectly coupled reaction sets (Co-sets) [74], have been calculated in genome-scale metabolic models. Co-sets are often along linear pathways [74]. Thus, a low co-morbidity of two metabolically linked diseases would indicate a missing link along a Co-set, which would break the flux coupling by creating a pathway split. Similarly, single nucleotide polymorphisms have been mapped onto metabolic networks [75] and may be used for identifying missing functions in human metabolism.

Ubiquitous unknowns e.g. genes with unknown function and orphan enzymes belonging to orthologous families, have been identified as top targets for functional elucidation in terms of biological knowledge payoff as these are ancient in origin and therefore likely to be involved in essential metabolic processes [22,25,76]. We believe that combining a metabolic network approach with knowledge of ubiquitous unknowns could also represent an ideal method for organism specific novel function identification.

## Conclusions

The results presented here show that RECON 1 allows the identification of specific metabolic pathways and reactions for which knowledge is lacking; thereby focusing the search for unknown metabolic functions by putting them into context with previously gathered metabolic information. Following identification and characterisation of RECON 1 network gaps, we showed that gap solution hypotheses can be generated automatically and successfully but require detailed, time consuming manual investigation in order to validate their biological plausibility. In this manner we have derived multiple hypotheses, which we intend to use to direct our knowledge-driven approach towards novel metabolic discoveries.

## Methods

### Pre-processing to gap identification

RECON 1 was obtained from Duarte et al. [11]. It contains 356 dead-end metabolites causing knowledge gaps in eight cellular compartments. The dead-end metabolites in the compartmentalised model are not unique, as the same metabolite can cause blocked reactions in two or more cellular compartments. In order to address this issue, RECON 1 was decompartmentalised, by placing all intracellular compartment reactions in the cytosol and removing duplicates. Extra-organism located reactions were kept. Therefore, the decompartmentalised network accounts for these two compartments. Subsequently, the number of dead-end metabolites was reduced to 145, as they were unique. Note that this modification affected the following gap analysis in that compartment specific gaps were not considered for gap filling; e.g., a reaction present in mitochondria but missing in cytosol would not result in a gap in the decompartmentalised network. All subsequent analysis was performed using the decompartmentalised version of RECON 1 (Recon_1_decomp).

As a next step, all blocked reactions present in Recon_1_decomp were identified using flux variability analysis (FVA) [27] as reactions unable to carry flux defined by |Vmax, i | ≤ 10^-5 ^mmol/g_dw_/hr and |Vmin, i |≤ 10^-5 ^mmol/g_dw_/hr for all i reactions in the network. All exchange reactions were unconstrained permitting free uptake and secretion of respective metabolites. A total of 285 reactions were identified.

The SMILEY algorithm has been described previously by Reed et al. [28] and implemented in the COBRA toolbox v2.0 (Schellenberger et al, submitted). We downloaded the KEGG [31] Ligand database (as of 1.10.2009), deemed **U**. Furthermore, we constructed a transport matrix, **X**, by defining a transport reaction from cytosol to extra-organism and an exchange reaction for each metabolite occurring Recon_1_decomp (**S**) and **U **(Figure 1). Matrix **U **and **X **served as reaction source for the SMILEY algorithm. Prior to the calculation, metabolites from **S **were matched to **U**. Note that not all metabolites in **S **have a known KEGG ID and that subsequent solutions are sensitive to missing KEGG IDs, meaning that some possible resolving solution may have been missed in our simulation due to this shortcoming. Current work has focused on adding more metabolite identifiers to RECON 1 (Thiele et al, in preparation).

In the next step, each blocked reaction v_b, i _was chosen as objective function, requiring the SMILEY algorithm to find reactions in **U **and/or **X **to be added to **S **such that |v_b, i_| could carry a flux greater than 10^-5 ^mmol/g_dw_/hr. SMILEY is designed to find the shortest possible solution consistent with this requirement [28]. For each blocked reaction, we computed the 20 shortest SMILEY solutions. Note that the number of solutions computed is user defined and in some cases 20 distinct solutions may not exist. For 175 out of 285 blocked reactions at least one SMILEY solution could be found.

### Gap analysis and validation of SMILEY output

SMILEY generated a total of 1335 solutions for the 175 blocked reactions. We filtered the solutions such that: i) only category II solutions with the least number of resolving reactions were investigated, and ii) if no category II solution was found, the solution involving the least number of resolving reactions was investigated. In cases where a blocked reaction had multiple solutions with the same number of resolving reactions, a random solution was picked. These criteria generated the S1 SMILEY solution output that is reported in the results.

Biochemical and genetic information concerning each blocked reaction identified by FVA was obtained from the Bigg database http://bigg.ucsd.edu/[18]. The reaction specific information allowed blocked reactions to be grouped depending on their metabolic pathway, subsystem, and/or other reaction specific features described within the Bigg database. Similarly, biochemical and genetic information concerning resolving reactions proposed by SMILEY was obtained from the KEGG database http://www.genome.jp/kegg/[31], which allowed organisation of the resolving reaction output. For resolving reactions where no human gene or protein information was directly available from Bigg or KEGG, blast homology searches of genes encoding the resolving reaction activity were performed against the human sequence databases on the NCBI website http://blast.ncbi.nlm.nih.gov/Blast.cgi and using the STRING database [77]. The localisation of dead-end metabolites in human biofluids was obtained from the human metabolome project http://www.hmdb.ca/[41]. Resolving reactions were investigated individually by literature review in order to verify the biological relevance of the proposed SMILEY solutions and generate plausible hypotheses for gap filling of RECON 1. Reaction directionalities were compared with experimentally reported reaction directionalities in the Brenda database [34] and by quantitative assignment of reaction directionality using the von Bertalanffy 1.0 algorithm [33] an extension available freely as part of the openCOBRA project [78]. The details of these calculations will be published in a separate manuscript. Briefly, experimentally determined or computed standard metabolite Gibbs energy transformed to cellular compartmental conditions with respect to *in vivo *pH (pH = 5.5-8.0), temperature (37°C), ionic strength (0.25 M) and electrical potential (-150 - 30 mV) was used to predict the upper and lower bounds on standard transformed reaction Gibbs energy. The upper and lower bounds are dependent upon *in vivo *metabolite concentration ranges which were set to 10^-7 ^- 10^-2 ^M. When the transformed reaction Gibbs energy range spans zero the reaction is predicted to be quantitatively reversible. Spontaneous reactions proposed by SMILEY were assessed in a similar manner as enzyme catalysed reactions. All gap filling hypotheses can be found in Additional files 6 and 7. Reaction and sub-cellular compartment abbreviations are as described in the BiGG database.

## Competing interests

The authors declare that they have no competing interests.

## Authors' contributions

OR carried out the experiments, analysed the data and wrote the manuscript. IT designed study, carried out experiments, analysed the data and contributed to the writing of the manuscript. BØP contributed to the design and writing of the manuscript. All authors read and approved the final manuscript.

## Supplementary Material

Additional file 1 **Metabolic subsystem distribution of the 175 blocked reactions**. The distribution of the blocked reactions identified in RECON 1 within metabolic subsystems. Click here for file

Additional file 2 **Dead-end metabolites can cause multiple blocked reactions**. In the majority of cases, dead-end metabolites only cause one blocked reaction. When a dead-end metabolite is at the end or beginning of a reaction cascade however, it inhibits flux through all reactions, which are part of the reaction cascade. The figure shows the number of blocked reactions found in the reaction cascades. For example, there is one reaction cascade, which has 14 blocked reactions. Click here for file

Additional file 3 **The SMILEY solution distribution is not dependent upon the metabolic pathway**. Blocked reactions can have multiple SMILEY solutions independent of the metabolic pathway, of which the blocked reaction is a part. Click here for file

Additional file 4 **Characterisation of alternative SMILEY solution**. The figure shows the solution categories of the alternative SMILEY solutions to blocked reactions, which had a category I (A), category II (B) or a category III (C) S1 solution. A) 30% of the blocked reactions with a category I S1 solution had alternative category III solutions. The remaining 70% had either an alternative category I solution or none at all. B) 61% of the blocked reactions, which had category II S1 solution, had alternative category II or III solutions. C) None of the blocked reactions, which had category, III S1 solutions had alternative category solutions as expected. Click here for file

Additional file 5 **The pathway distribution of blocked reactions and their SMILEY S1 solutions categories**. The figure shows the SMILEY S1 solution category as a percentage of the total number of blocked reactions found within a particular metabolic pathway. The number of blocked reactions within each metabolic pathway and their SMILEY solution type is also shown. Some blocked reactions, such as those involved in amino acid metabolism, are easily bypassed using functionalities already described in the KEGG, represented by category I and II SMILEY solutions. Others, such as those involved in glycan biosynthesis, can only be solved by transport of their causative dead-end metabolite out of the system. Click here for file

Additional file 6 **The filtered S1 SMILEY output**. The table contains the filtered SMILEY S1 output as described in materials and methods. The identified blocked reaction is given in column A. Columns B-J report properties of the blocked reactions i.e., causative dead end metabolites, BiGG confidence scores, cellular compartment, whether or not the blocked reaction is in a cascade, blocked reaction components, associated genes, the metabolic subsystem and the metabolic pathway the blocked reaction occurs in. Columns K-O report properties of the S1 solution i.e. the solution category, the experimentally reported directionality and computed directionalities of blocked reactions having category I reversal solutions, the number of resolving reactions composing the solution and the KEGG identifiers of each resolving reaction. The remaining columns report properties of each individual resolving reaction comprising the SMILEY solution i.e. KEGG identifiers, resolving reaction components, metabolic pathway and subsystem and any associated enzyme commission numbers or known human genes. Click here for file

Additional file 7 **The complete SMILEY output**. The table contains the complete SMILEY output with up to twenty solutions for each identified blocked reaction. The identified blocked reaction is given in column A. In columns B-D the properties of the blocked reaction are reported (reaction components, gene and metabolic subsystem). In column E the resolving reactions comprising the complete SMILEY solution for the blocked reaction is reported with KEGG reaction identifiers. In the following columns the properties of the resolving reactions are reported (reaction components, reaction subsystem, enzyme commission number and gene). Click here for file
