# Supplementary figures and images for: The human metabolic reconstruction Recon 1 directs hypotheses of novel human metabolic functions
Source: BMC Syst Biol. 2011 Oct 1;5:155. doi: 10.1186/1752-0509-5-155 (PMC3224382; doi:10.1186/1752-0509-5-155)

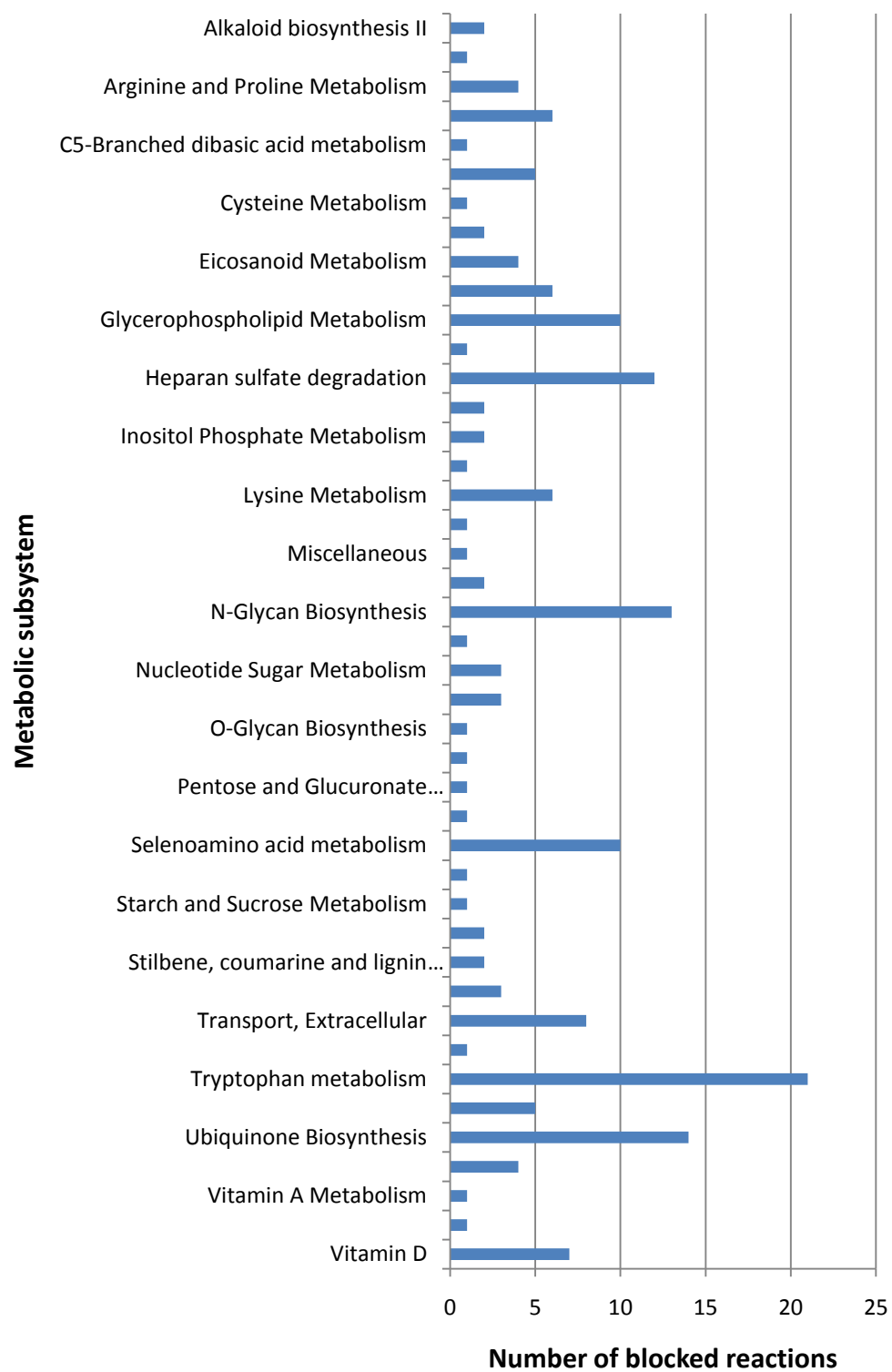

Supplement: Additional file 1 — Metabolic subsystem distribution of the 175 blocked reactions. The distribution of the blocked reactions identified in RECON 1 within metabolic subsystems. [file 1752-0509-5-155-S1.PDF]

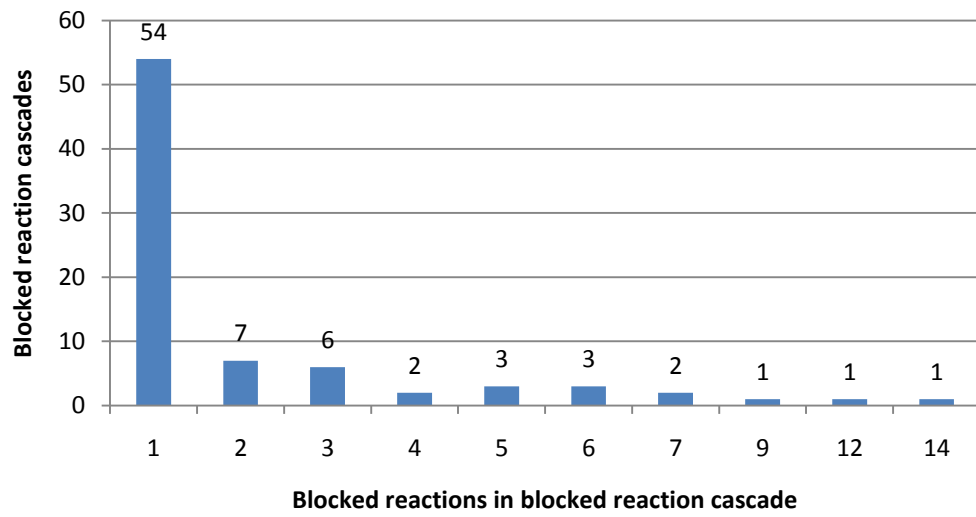

Supplement: Additional file 2 — Dead-end metabolites can cause multiple blocked reactions. In the majority of cases, dead-end metabolites only cause one blocked reaction. When a dead-end metabolite is at the end or beginning of a reaction cascade however, it inhibits flux through all reactions, which are part of the reaction cascade. The figure shows the number of blocked reactions found in the reaction cascades. For example, there is one reaction cascade, which has 14 blocked reactions. [file 1752-0509-5-155-S2.PDF]

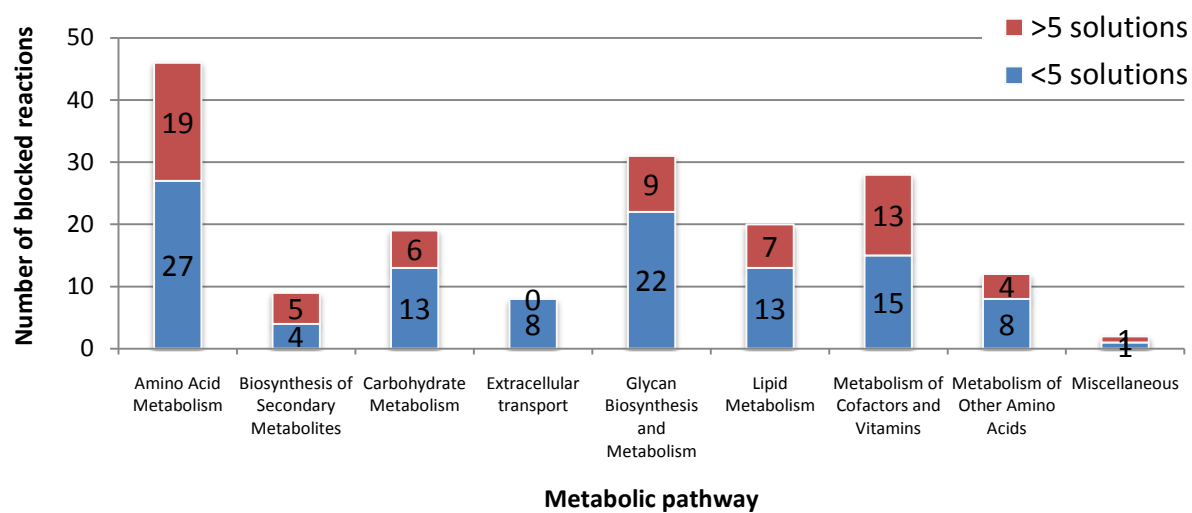

Supplement: Additional file 3 — The SMILEY solution distribution is not dependent upon the metabolic pathway. Blocked reactions can have multiple SMILEY solutions independent of the metabolic pathway, of which the blocked reaction is a part. [file 1752-0509-5-155-S3.PDF]

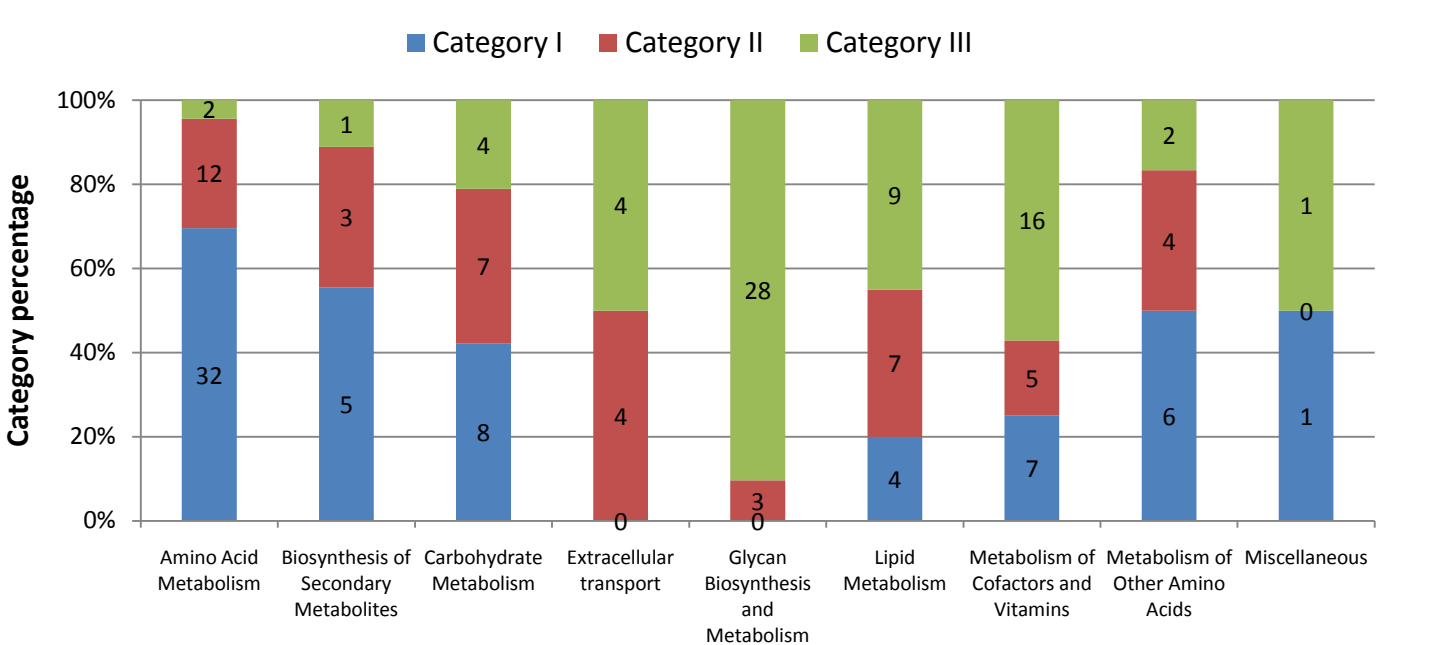

Supplement: Additional file 5 — The pathway distribution of blocked reactions and their SMILEY S1 solutions categories. The figure shows the SMILEY S1 solution category as a percentage of the total number of blocked reactions found within a particular metabolic pathway. The number of blocked reactions within each metabolic pathway and their SMILEY solution type is also shown. Some blocked reactions, such as those involved in amino acid metabolism, are easily bypassed using functionalities already described in the KEGG, represented by category I and II SMILEY solutions. Others, such as those involved in glycan biosynthesis, can only be solved by transport of their causative dead-end metabolite out of the system. [file 1752-0509-5-155-S5.PDF]
