# Supplementary material for: The human metabolic reconstruction Recon 1 directs hypotheses of novel human metabolic functions
Source: BMC Syst Biol. 2011 Oct 1;5:155. doi: 10.1186/1752-0509-5-155 (PMC3224382; doi:10.1186/1752-0509-5-155)
Supplement: Additional file 4 — Characterisation of alternative SMILEY solution. The figure shows the solution categories of the alternative SMILEY solutions to blocked reactions, which had a category I (A), category II (B) or a category III (C) S1 solution. A) 30% of the blocked reactions with a category I S1 solution had alternative category III solutions. The remaining 70% had either an alternative category I solution or none at all. B) 61% of the blocked reactions, which had category II S1 solution, had alternative category II or III solutions. C) None of the blocked reactions, which had category, III S1 solutions had alternative category solutions as expected. [file 1752-0509-5-155-S4.PDF]

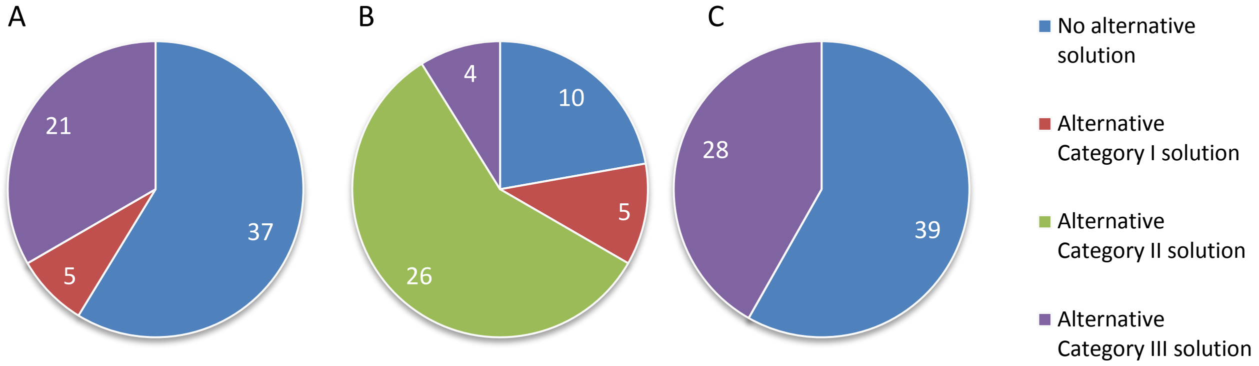
A
B
C
37
5
21
10
5
26
4
39
28
No alternative solution
Alternative Category I solution
Alternative Category II solution
Alternative Category III solution
